# Tick-borne encephalitis: from tick surveillance to the first confirmed human cases, the United Kingdom, 2015 to 2023

**DOI:** 10.2807/1560-7917.ES.2025.30.5.2400404

**Published:** 2025-02-06

**Authors:** Helen Callaby, Kate R Beard, Dominic Wakerley, Mary Alexandra Lake, Jane Osborne, Kevin Brown, Nadina Wand, Jenny Warner, Maya Holding, Nicholas WS Davies, Malcolm Proudfoot, Amanda Semper, Tim Brooks, Christina Petridou, Catherine F Houlihan, Tommy Rampling, Clare E Warrell, N Claire Gordon, the TBE working group, TBE working group, Christopher Thorne, Robin Brittain-Long, Fahed Bangash, Adam Stafford, Carolin Bresges, Jonathan Underwood, Samir Dervisevic, Jonathan Graham, Monique I Andersson

**Affiliations:** 1 United Kingdom Health Security Agency (UKHSA) Rare and Imported Pathogens Laboratory, Porton Down, Salisbury, United Kingdom; 2 University of Aberdeen, Aberdeen, United Kingdom; 3 Hampshire Hospitals National Health Service (NHS) Foundation Trust, Hampshire, United Kingdom; 4 University College London Hospital, London, United Kingdom; 5 United Kingdom Health Security Agency (UKHSA) Virus Reference Department, Colindale, United Kingdom; 6 Nuffield Department of Medicine, University of Oxford, Oxford, United Kingdom; 7 United Kingdom Health Security Agency (UKHSA) Diagnostics and Pathogen Characterisation Division, Porton Down, Salisbury, United Kingdom; 8 Imperial College Healthcare National Health Service (NHS) Trust, London, United Kingdom; 9 York and Scarborough Teaching Hospitals, York, United Kingdom; 10 London School of Hygiene and Tropical Medicine, London, United Kingdom; 11 National Institute for Health and Care Research (NIHR) University College London Hospitals Biomedical Research Centre (BRC), London, United Kingdom; 12 The members of the TBE working group are listed under Collaborators

**Keywords:** Tick-borne encephalitis, Vector-borne disease

## Abstract

**Background:**

Tick-borne encephalitis virus (TBEV) is a flavivirus spread by ticks and can cause tick-borne encephalitis (TBE) in humans. Previously, TBE has been reported in returning travellers in the United Kingdom (UK), but in 2019 and 2020, two probable cases of TBE acquired in the UK were identified.

**Aim:**

The aim of this study was to investigate TBE cases in the UK between 2015 and 2023, describing the incidence, place and mode of acquisition and diagnostic process.

**Methods:**

A retrospective review of possible, probable and confirmed cases of TBE diagnosed by the Rare and Imported Pathogens Laboratory (RIPL) between January 2015 and December 2023 was performed. For cases identified in 2022 and 2023, clinical data were collected for enhanced surveillance using structured case record forms. Laboratory diagnosis is reviewed and described.

**Results:**

We identified 21 cases: three possible, seven probable and 11 confirmed cases. Of these, 12 were between January 2022 and December 2023: three possible, three probable and six confirmed cases. Two confirmed TBE cases had definite or highly probable acquisition in the UK, in June and August 2022, respectively. One of the possible cases had definite UK acquisition. Cases typically have a biphasic presentation, with encephalitis in the second phase.

**Conclusion:**

Clinicians should be aware of the possibility of TBE when the cause for encephalitis is not identified, even in the absence of travel to previously identified endemic regions.

**Key public health message**

**What did you want to address in this study and why?**

Up until now, it was not thought that you could catch tick-borne encephalitis (TBE) in the United Kingdom (UK). Over recent years, we have learnt first that tick-borne encephalitis virus (TBEV) exists in ticks and other animals in the UK. We now describe how humans have acquired it in this country, how they present and how they are diagnosed.

**What have we learnt from this study?**

Between 2015 and 2023, 21 TBE cases with a clinical disease were diagnosed in the UK. Of these, 12 were diagnosed 2022–2023. Three cases acquired the infection in the UK which further demonstrates how important it is to have surveillance programmes.

**What are the implications of your findings for public health?**

Doctors should now consider whether TBE could be the cause in patients who present with unexplained encephalitis. Current public health campaigns encourage everyone to be tick aware with the aim to try and prevent Lyme disease transmission. These campaigns should also now include TBEV.

## Introduction

Tick-borne encephalitis virus (TBEV) is a flavivirus spread to humans and animals mainly by bites from the *Ixodes ricinus* and *Ixodes persulcatus* ticks [1]. Consumption of unpasteurised milk can sometimes lead to the acquisition of tick-borne encephalitis (TBE), but this mode of transmission is considered rare [1]. The TBEV complex is a serocomplex of viruses that includes TBEV and other related viruses, such as louping ill virus (LIV) and Powassan virus [2].

The incubation period for TBE, the clinical syndrome caused by TBEV, is 7–14 days, with a maximum of 28 days. An estimated two-thirds of human infections with TBEV are asymptomatic [3,4]. Symptomatic illness is often biphasic, with initial non-specific complaints including headache and fatigue. After a short recovery, a minority of patients progress to neurological disease including meningo-encephalitis [5].

There is no specific antiviral treatment available for TBE. Management is supportive, and critical care admission may be required in severe cases. Vaccines against TBEV are available and have been shown to be effective [4-6].

Tick-borne encephalitis virus is endemic in northern and eastern Asia, eastern, central and northern Europe, with new hotspots also emerging in some western European countries [7,8]. The primary vector for TBEV in the United Kingdom (UK), *Ixodes ricinus*, was already established across the British Isles, but there has been an observed increase in the number of reported bites from 2013 to 2020 [9]. Although reporting bias may be a contributing factor, climate change may also be partly responsible.

Previously, TBEV was considered absent from the UK, although LIV is endemic in the UK, causing disease in sheep, cattle and other livestock and occasional human infections [7]. Recognising the potential risk for TBEV ingress into the UK, systematic surveillance was conducted in ticks and sentinel animals, such as deer [8]. The surveillance programme tested for TBEV antibodies in culled deer in England and Scotland, and results were used to identify areas for focused tick collection. Foci of TBEV were identified in the East of England (Thetford Forest area) and in southern England (Hampshire/Dorset border) [10,11], with presence of virus in ticks confirmed by PCR. This provided evidence that TBEV is present in the UK in established enzootic cycles.

Subsequently, two probable human cases were identified in 2019 and 2020 based on serological testing [12,13]. Both cases had sustained tick bites in the Hampshire area in the south of England, and had compatible clinical syndromes, positive serology and no significant overseas exposure. However, virus was not detected on PCR testing, and the possibility of a similar TBE complex virus such as LIV could not be excluded.

Testing for TBE in the UK is indicated only when a patient has compatible clinical illness and relevant exposure (usually including travel to an endemic country), although since 2023, TBE testing is now done on all referred undiagnosed encephalitis cases even if no known exposure to ticks or travel. In this study, we performed a retrospective analysis of the TBE cases diagnosed in the UK.

## Methods

### Background to testing in the United Kingdom

The UK Health Security Agency (UKHSA) Rare and Imported Pathogens Laboratory (RIPL) [14] is the only clinical diagnostic laboratory in the UK to perform diagnostic testing for TBEV. At RIPL, PCR testing is available on serum, urine and cerebrospinal fluid (CSF), and IgG antibody testing on serum and CSF. In some other countries, TBEV PCR is used for diagnosis, and it is one of the laboratory criteria for diagnosis recommended by European Centre for Disease Prevention and Control (ECDC) [15]. The TBEV PCR used at RIPL is an in-house real-time assay developed from a published method which is fully accredited for clinical diagnostic use [16]. The TBEV PCR detects both TBEV and LIV, and a second specific PCR for LIV (in-house assay for research use only) is used to differentiate these two viruses. For antibody testing, TBEV IgG is performed using a commercial immunofluorescence assay (Euroimmun, Lübeck, Germany).

Based on the surveillance data and the possible cases, RIPL now tests for TBEV as part of routine clinical testing in samples referred from patients with a relevant clinical and tick exposure history regardless of travel history, which would previously have been considered a necessary risk factor for TBEV acquisition. All results are reviewed by a member of the RIPL clinical team and interpreted with reference to clinical, vaccination and exposure history and in the context of other available results.

This paper describes all cases of TBE diagnosed in the UK between 2022 and 2023 following the enhanced testing approach, including two cases of PCR-positive TBEV in patients without compatible travel history for imported infection, representing the first confirmed autochthonous cases of human TBE in the UK. Of note, TBE is not currently notifiable in the UK.

### Indication for the study

**Figure 1:**
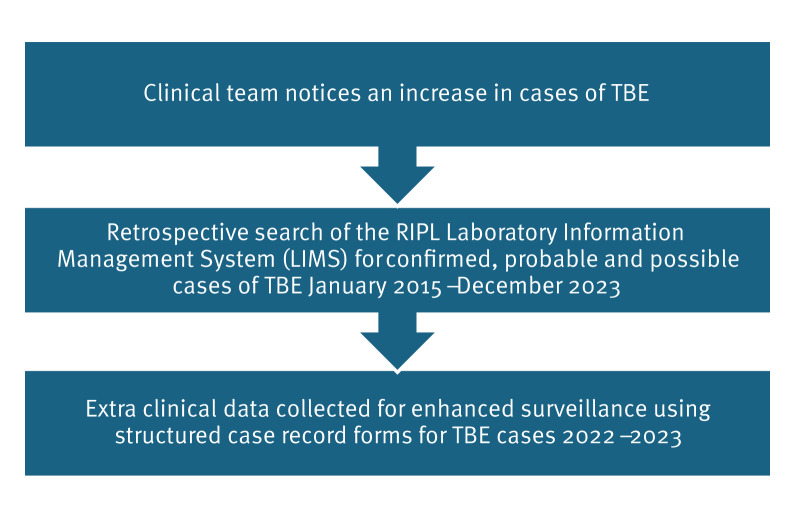
Study flowchart of investigation of diagnosed cases of tick-borne encephalitis, the United Kingdom, 2022–2023

RIPL: The UK Health Security Agency Rare and Imported Pathogens Laboratory; TBE: tick-borne encephalitis.

An observed increase in the frequency of positive TBE diagnoses throughout 2022 prompted an enhanced review of all cases diagnosed between 2022 and 2023 (Figure 1).

### Retrospective search

A retrospective search of the RIPL Laboratory Information Management System (LIMS) was performed for possible, probable and confirmed cases of TBE between January 2015 and December 2023, based on PCR and IgG testing. For cases identified in 2022 and 2023, clinical data were collected for enhanced surveillance using structured case record forms sent to the referring clinical team. The questionnaire is available in the Supplement.

### Categorisation of cases

Cases were categorised as possible, probable or confirmed TBE based on the criteria outlined (Box), following review of the case record form and available laboratory results.

Box **Definition of possible, probable and confirmed cases of tick-borne encephalitis (TBE), the United Kingdom**

**Possible case of TBE:**

• Compatible clinical syndrome (symptoms of central nervous system inflammation, such as meningitis, meningo-encephalitis, encephalomyelitis, encephaloradiculitis)

AND

• Detection of IgG antibodies in single unpaired serum sample where this cannot be explained by previous antigen exposure.

**Probable case of TBE:**

• Compatible clinical syndrome

AND

• Rise in TBEV IgG antibody titre but which does not meet definition of confirmed case

OR

• Evidence of intrathecal antibody production of IgG to TBEV.

**Confirmed case of TBE:**

• Compatible clinical syndrome

AND

• Detection of TBEV complex RNA in blood, urine or CSF

OR

• ≥ 4-fold increase of TBEV IgG antibodies in paired serum samples.

CSF: cerebrospinal fluid; TBEV: tick-borne encephalitis virus.

Cases with PCR-positive test results were also tested using the LIV-specific assay, which is more sensitive and specific for LIV than the TBEV PCR. Cases, which were TBEV complex PCR positive and LIV PCR negative, were designated as confirmed TBE cases.

Where CSF IgG testing was performed and was positive, total intrathecal antibody levels were also measured to determine whether the IgG present in the CSF was due to increased permeability of the blood–brain barrier or active intrathecal antibody production.

Where confirmed TBEV cases were identified, with acquisition likely to be in the UK, the samples were sent for next generation whole genome sequencing.

## Results

### Retrospective search 2015–2023

In the LIMS search, we identified 21 cases with either positive molecular or serological testing leading to a final coded diagnosis of possible, probable and confirmed TBE between 2015 and 2023 (Table).

**Table:** Possible, probable and confirmed cases of tick-borne encephalitis diagnosed at the UK Health Security Agency Rare and Imported Pathogens Laboratory (RIPL), the United Kingdom, 2015–2023 (n = 21)

Case category	2015	2016	2017	2018	2019	2020	2021	2022	2023
Possible	0	0	0	0	0	0	0	2	1
Probable	0	1	0	1	0^a^	2	0	2	1
Confirmed	0	1	0	2	2	0	0	4	2
Total	0	2	0	3	2	2	0	8	4

LIMS: Laboratory Information Management System; TBEV: tick-borne encephalitis virus; UK: United Kingdom.

^a^ A probable case of TBEV with UK acquisition was diagnosed in Germany in 2019 [12] and is not included in this table as it was not in the RIPL LIMS system to appear in the data search.

### Enhanced surveillance

Between January 2022 and December 2023, 12 TBE cases were diagnosed: three possible, three probable and six confirmed, per the definitions above (Box). Of the six confirmed cases, three were PCR-positive on a CSF sample, (quantification cycle (Cq) values: 38.3, 37.3, 36.6), two were PCR-positive on serum (Cq: 36.3 and 34.4) and one case had > 4-fold rise in IgG titre on sequential samples. Results are summarised in Supplementary Table 2.

It should be noted that the LIMS codings were not made according to the definitions described in the methods, and a retrospective analysis of the 2022 and 2023 cases using the newer categorisation criteria culminated in the final diagnosis as listed in Supplementary Table 2.

The median age of the cases was 50 years (range: 8–67 years), with nine males and three females. Nine patients reported travel within a timeframe (within 28 days) compatible with TBEV acquisition outside the UK. All travel was within Europe. Five patients had travelled to Sweden, two to Germany, one to Lithuania and one to Poland. Two of the confirmed TBE cases had definite (Case 2) or highly probable (Case 6) acquisition in the UK, in June and August 2022, respectively. One of the possible cases had definite UK acquisition (Case 12). More information of the cases is presented in Supplementary Tables 2 and 3.

Nine patients reported an observed tick bite, and one reported multiple possible insect bites but was unsure if they had been exposed to ticks. Of the seven 2022 cases with definite tick bites, the mean time from bite to initial symptom onset was 4.5 days (range: 2–14 days). Of all 12 cases 2022–2023, ten reported a biphasic illness. Eleven reported a headache, and eight reported fevers. Ten patients had neurological symptoms, which included primarily ataxia and diplopia. Confusion was also reported in three patients.

### Cases infected in the United Kingdom

**Figure 2:**
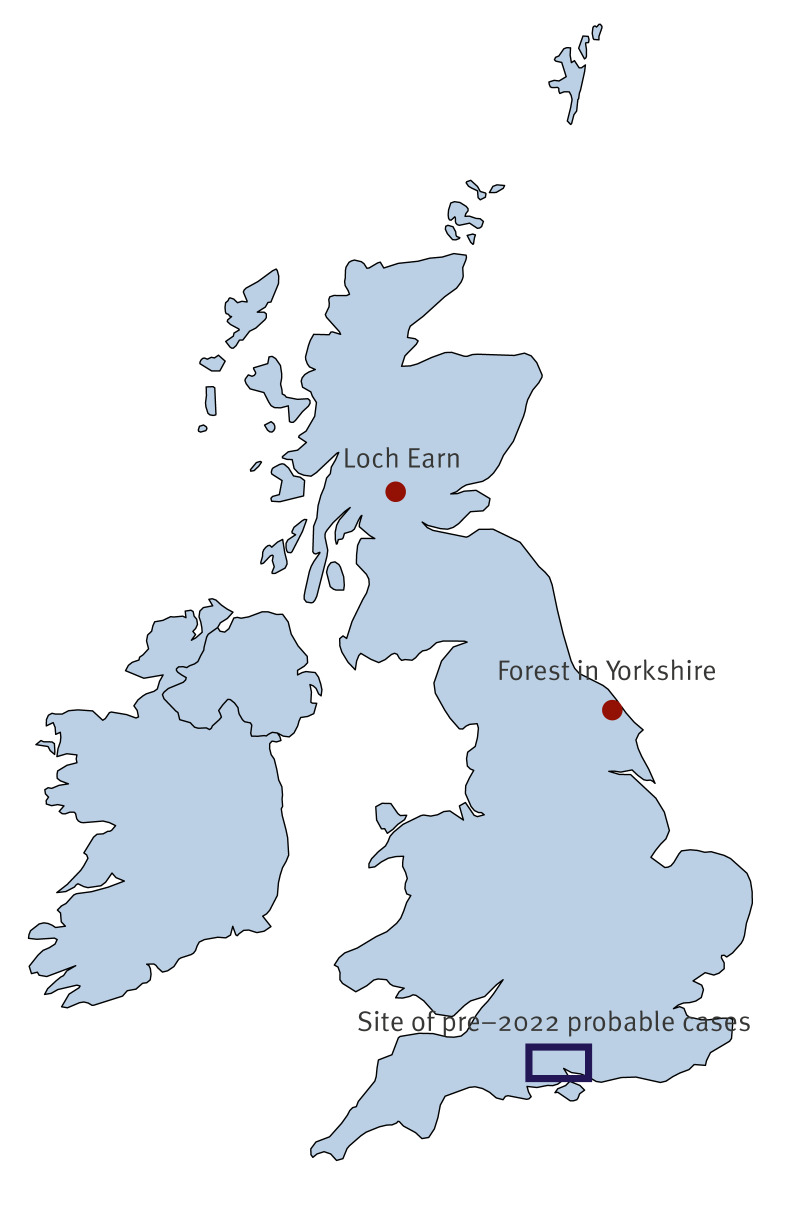
Map presenting place of infection of autochthonous tick-borne encephalitis cases, the United Kingdom, 2019–2023 (n = 4)

Of the two confirmed UK-acquired cases (Cases 2 and 6), Case 2 was a 28-year-old male who sustained multiple tick bites while walking near Loch Earn in Scotland (Figure 2). Two days after the bites, he reported a mild, nonspecific illness with fatigue, which lasted 24 h. Twelve days later, he developed neck stiffness, photophobia and frontal and bitemporal headache. His symptoms progressed, and he presented to his local emergency department several days later when he developed diplopia. On examination, he had an ataxic gait, diplopia on rightward gaze and gaze-evoked nystagmus worse on looking to the right. Lumbar puncture demonstrated 106 white cells/mm^3^ (96% mononuclear lymphocytes) in the CSF. Testing of the CSF was negative for herpes simplex virus (HSV), varicella-zoster virus (VZV) and enterovirus. Samples were sent to RIPL for further testing due to the tick exposure, and a diagnosis of TBE was made based on the positive TBEV PCR (Cq: 38.3) of CSF and accompanying rise in serum anti-TBEV IgG, LIV PCR of CSF was negative. Detailed results are presented in Supplementary Table 2. Treatment was supportive with analgesia and fluids. He made a gradual recovery, however, at follow-up 8 weeks after symptom onset, he still had mild intermittent headache, poor balance hindering sporting activities and some residual dizziness.

Red dots present the location of transmission for two confirmed cases.

Case 6 was a 50-year-old male who sustained a tick bite while mountain biking in a forest in Yorkshire in England (Figure 2). One day before he presented, he had returned from a holiday in Kos, Greece, where he had undertaken multiple outdoor activities, but had not noted any insect bites nor consumed raw milk products. On day 7 post tick bite, he developed fevers, myalgia, severe fatigue and poor sleep. On day 10, he was admitted to hospital and received antibiotics for undifferentiated infection. Blood tests at this point were unremarkable except for neutropenia, with a C-reactive protein (CRP) of < 1 mg/L. His symptoms resolved over the following week, and he was discharged, however, on day 23 post exposure his symptoms relapsed, and he was readmitted with fevers, abdominal pain and headache. Examination demonstrated mild cerebellar ataxia with gait imbalance. Analysis of CSF showed 40 white cells/mm^3^ (97% lymphocytes). Standard bacterial culture was negative, and PCR testing was negative for HSV, VZV and enterovirus. Serum and CSF samples were sent to RIPL for TBEV testing, including a follow-up serum sample. Serum from the admission was PCR-positive for TBEV complex (Cq: 34.4), accompanied by an increase in serum anti-TBEV IgG titre from 1:320 to 1:10,000 in a follow-up sample taken 10 days after the initial. Result of TBEV PCR on CSF was negative, however, he was found to have intrathecal production of anti-TBEV IgG. Testing with LIV PCR on serum resulted negative.

By day 29, his fever had resolved, and he was discharged on day 32. At follow-up 2 weeks later, all neurological signs had resolved. Tick-borne encephalitis virus is not known to be endemic in Greece and is not identified as a risk in the region visited, and therefore it is considered highly likely that the infection was acquired in the UK.

Next generation whole genome sequencing was attempted on samples from both cases, but did not yield a result due to the high Cq values.

## Discussion

This paper confirms domestically acquired TBEV infection in the UK, reporting the first PCR-confirmed case with no history of foreign travel and a second confirmed case with definite exposure to domestic ticks. The reasons for the emergence of TBEV in the UK are not fully established, although it is likely that multiple factors are involved. Climate change is one suggestion, yet the varied climate predilections of tick species introduce complexities in drawing associations with climate change. *Ixodes ricinus* is notable in having an already wide geographic distribution and a rise in global temperatures would likely only broaden its climate range [17,18]. Climate change could impact the tick proliferation rate, duration of questing season (and hence transmission) and human behaviour among other potential factors [18]. Previous modelling of how a warming climate would impact the risk of Lyme disease — also transmitted by *I. ricinus* — predicted greater risk of transmission across Scotland [19].

Most TBEV infections in humans are asymptomatic or pauci-symptomatic, so they may remain undetected [20]. In countries where TBEV has recently emerged, numbers of diagnosed cases are low. For example, the Netherlands, which reported its first autochthonous case in 2016 [21], reported only five cases in total in 2020 [22] and a total of 21 cases to date [23,24]. Across the European Union between 2012 and 2016, there were 12,500 neurologically symptomatic cases in total reported [25]. Of the 9,889 cases with outcome data available, there were 48 deaths (0.5%) and 247 (2.5%) individuals left with neurological sequelae after 5 years [22].

Diagnosis of TBE relies on correct timing of sampling. Patients may have mild symptoms during the initial viraemic phase, which is important to consider when performing molecular testing, as the virus may be undetectable by PCR at the time the patient presents with symptoms. Cross-reactivity in IgG with other flaviviruses is also seen and may complicate diagnosis. It is therefore important to obtain an accurate history, including vaccination and exposure to other flavivirus infections, in order to interpret serology correctly. Yellow fever, dengue, Japanese encephalitis and TBEV vaccination are likely to affect results. The presence of LIV in the UK further complicates the interpretation of results, and additional testing by LIV-specific PCR, along with surveillance information from animal health authorities, is required to determine the likelihood of TBEV infection vs LIV. In the UK, there have been no confirmed human infections with LIV for over 25 years [7].

All patients described here had neurological symptoms. It is likely there is underdiagnosis and underreporting of TBE, particularly with a large proportion of patients being asymptomatic or with mild symptoms.

Alongside the headline findings of UK-acquired TBEV infection, this case series also demonstrates a small rise in the number of imported TBE cases to the UK. All these cases were acquired in Europe during spring and summer, and almost all had a history of recent tick bites. Notably, there were no cases from other TBEV-endemic areas such as northern and eastern Asia. The risk of TBEV should be considered before travel; none of these cases had a confirmed history of TBEV vaccination even when travelling to an area noted for high endemicity.

In this example, awareness of the evolving epidemiology of TBEV in Europe, raised through international surveillance and reporting through ECDC, triggered the reservoir and tick sampling programme which confirmed the presence of TBEV in the UK. These data were then used to inform the diagnostic testing approach for suspected cases of infectious encephalitis referred to the RIPL. This demonstrates the importance of a One Health approach with communication between animal and vector disease surveillance and clinicians. Had there not been an awareness of exposure of deer to TBEV and presence in ticks within the UK, TBEV would not have been considered as a diagnosis in these patients.

Subsequently, press coverage and proceedings at national conferences and educational events has sought to increase awareness of the possibility of TBEV infections among physicians regarding TBE symptoms and diagnosis, and to encourage them to suspect TBEV infection in patients presenting with unexplained encephalitis and a plausible exposure history including within the UK.

## Conclusion

Autochthonous human TBE has been diagnosed in the UK, in two separate geographic locations. Tick surveillance studies correlate with the diagnosis of human cases. This study demonstrates an exemplar relationship between international and national surveillance programmes, clinical diagnosis and awareness raising among clinicians.

Further surveillance of ticks and sentinel animals is indicated across larger areas of the UK, and public health alerts have been released to ask clinicians to consider TBEV as a diagnosis in unexplained encephalitis. Further surveillance work is suggested, including retrospective testing of CSF samples in patients with have encephalitis not explained by another cause.

## Supplementary Data

806000 Supplementary Material
