# Supplementary material for: Tick-borne encephalitis: from tick surveillance to the first confirmed human cases, the United Kingdom, 2015 to 2023
Source: Euro Surveill. 2025 Feb 6;30(5):2400404. doi: 10.2807/1560-7917.ES.2025.30.5.2400404 (PMC11803743; doi:10.2807/1560-7917.ES.2025.30.5.2400404)
Supplement: Supplementary Material [file 24-00404_CALLABY_Supplement.pdf]

This supplementary material is hosted by Eurosurveillance as supporting information alongside the article **"Tick-borne encephalitis: from tick surveillance to the first confirmed human cases, the United Kingdom, 2015 to 2023",** on behalf of the authors, who remain responsible for the accuracy and appropriateness of the content. The same standards for ethics, copyright, attributions and permissions as for the article apply. Supplements are not edited by Eurosurveillance and the journal is not responsible for the maintenance of any links or email addresses provided therein.

Table 2: Laboratory results used to inform diagnosis for those with confirmed, probable and possible TBEV in the UK 2022 and 2023.

**Case**	**1**	**2**	**3**	**4**	**5**	**6**	**7**	**8**	**9**	**10**	**11**	**12**
**CSF TBEV PCR**	No sample sent	Positive (Ct 38.26) Confirmed on repeat testing	Negative	Negative	No sample sent	Negative	Indeter-minate (CT 37.14 but could not be confirmed on repeat testing)	Negative	Positive (Ct 37.3) Confirmed on repeat testing	Positive (Ct 36.6) Confirmed on repeat testing	Negative	Negative
**CSF Louping Ill PCR**	n/a	Negative	n/a	n/a	n/a	n/a	Negative	n/a	Not done - acquired in country with no LIV	Not done - acquired in country with no LIV	n/a	Negative
**Serum TBEV PCR**	Negative	Negative	Negative	Negative	Positive (Ct 36.4) Confirmed on repeat testing	Positive (CT 34.4) Confirmed on repeat testing	Negative	Negative	Negative	Negative	Negative	Negative
**Serum Louping Ill PCR**	Negative	n/a	n/a	n/a	Negative	Negative	Negative	n/a	n/a	n/a	n/a	Negative
**Urine TBEV PCR**	No sample sent	No sample sent	Negative	No sample sent	No sample sent	Negative	Negative	Negative	No sample sent	Negative	No sample sent	No sample sent
**Serum TBEV IgG titre 1**	>1:10,000	1:100	1:3200	1:1000	1:10,000	1:3200	1:320	1:10,000	1:1000	1:1000	1:10,000	1:10
**Serum TBEV IgG titre 2**	No sample sent	1:3200	1:10,000	No sample sent	No sample sent	1:10,000	1:3200	1:10,000	1:10,000	>1:10,000	No sample sent	>1:10,000
**CSF TBEV IgG titre 1**	n/a	Not done as PCR positive	1:100	1:32	n/a	1:100	Positive neat only	1:100	1:32	1:32	1:1000	1:10
**CSF TBEV IgG titre 2**	n/a	n/a	Not done	Not done	n/a	Not done	Not done	Not done	Not done	1:1000	Not done	Not done
**CSF Intrathecal TBEV antibodies**	n/a	n/a	Blood-brain barrier dysfunction - no evidence of intrathecal antibody production	Blood-brain barrier dysfunction - no evidence of intrathecal antibody production	n/a	n/a	Not done due to low IgG titre	Blood-brain barrier dysfunction - no evidence of intrathecal antibody production	Not done	Not done	Not done	Not done
**Diagnosis**	Possible TBEV: compatible syndrome, unvaccinated, positive IgG but no follow up sample	Confirmed TBEV: PCR positive	Probable TBEV: increase in serum IgG	Possible TBEV: compatible syndrome, unvaccinated, positive IgG but no follow up sample	Confirmed TBEV: PCR positive	Confirmed TBEV: PCR positive	Confirmed TBEV: significant rise in serum IgG with PCR at limit of detection	Probable TBEV	Confirmed TBEV: PCR positive	Confirmed TBEV: PCR positive	Probable TBEV: raised serum IgG with likely intrathecal antibody production	Possible TBEV

**Flavivirus vaccination history**	Not vaccinated	Not vaccinated	Yellow Fever vaccination >30 years ago	Not vaccinated	Not known	Yellow fever vaccine >30 years ago	Not vaccinated	Not known	Not vaccinated	Not known	Not vaccinated	Not vaccinated
**Other information**			Dengue IgG and Yellow Fever IgG also detectable					As the TBEV serum IgG titre was at the upper limit of the assay on both occasions, a rise in titre cannot to be confirmed.			CSF IgG tested in parallel with a serum sample. High titre of IgG to TBE virus in CSF suggestive of recent infection although blood-brain barrier dysfunction not excluded.	LIV PCR on serum and CSF performed as no recent travel outside UK

Table 3: Clinical Features of those diagnosed with for those with confirmed, probable and possible TBEV in the UK 2022 and 2023

**Patient**	**1**	**2**	**3**	**4**	**5**	**6**	**7**	**8**	**9**	**10**	**11**	**12**
**Age**	50-60	20-30	40-50	50-60	60-70	50-60	50-60	20-30	60-70	40-50	50-60	0-10
**Sex**	M	M	M	M	F	M	M	F	M	F	M	M
**Travel History**	Y	N	Y	Y	Y	Y	Y	Y	Y	Y	Y	N
**Country**	Sweden	Scotland	Sweden	Bavaria and Italy	Sweden	Kos	Poland	Lithuania	Germany	Sweden	Sweden	UK
**Reports tick bite(s)?**	Y	Y	Y	Y	Y	Y	Y	N	Y	N	Y	N
**Biphasic Illness**	Y	Y	Y	Y	Y	Y	N	Y	N	Y	Y	Y
**Headache**	Y	Y	Y	Y	Y	Y	Y	Y	N	Y	Y	Y
**Fever**	Y	Y	Y	N	N	Y	Y	Y	N	N	Y	Y
**Neurological findings**	Diplopia, ataxia	Diplopia, ataxia, photophobia	Diplopia, ataxia, confusion	None	Ataxia	Ataxia	Ptosis, proximal weakness	None	Bulbar palsy, confusion	Right arm weakness, confusion	Meningism and photophobia	Photophobia
**Rash**	Y	N	N	N	Y	N	N	N	N	N	N	Y
**CT findings**	Indirect evidence of raised ICP	No acute intracranial findings	No acute intracrania l findings	No acute intra-cranial findings	Possible recent right internal capsule infarct	No acute intracranial findings	No acute intracranial findings	No acute intra-cranial findings	Hyper-tensive micro-angiopathy with emboli	No information	No information	Not done
**MRI findings**	Not done	Inflammatory changes cerebellum and brainstem, with lepto-meningeal enhancement	Not done	Not done	No evidence acute infarct or haemo-rrhage	Cerebellar vermis signal change	No acute intracranial findings	No acute intra-cranial findings	Hyper-tensive micro-angiopathy	No information	No information	Not done
**EEG**	Not done	Not done	Not done	Not done	Mild diffuse cerebral dys-function with some focal areas	Not done	Non-specific changes could be in keeping with viral encephalitis or other aetiologies.	Not done	Not done	Not done	Not done	Not done

**CSF leucocyte count / mm^3)**	148 (95% lympho-cytes)	102 (96% lymphocytes)	115 (100% lympho-cytes)	12 (no differential count)	12 (no differ-ential count)	40 (97% lympho-cytes)	1160 (60% lympho-cytes)	140 (83% lympho-cytes)	110 (95% lymphocytes)	120 (differential not provided)	No information	176 (99% lympho-cytes)

Structured case record forms for enhanced surveillance sent to 2022 cases

TBE Case

UK Health Security Agency

Dear whom it may concern

Within the Rare and Imported Pathogens Laboratory (RIPL), we are investigating a possible/probable case of TBE which you referred to us.

Patient Name:

DOB:

Age:

Gender:

In order to help us interpret the serology, we wonder if you could provide the following information:

Travel history (exact location):

Travel dates:

Tick bite (& dates):

Country where bitten by tick:

Biphasic illness: Y/N

Clinical history:

Date of symptom onset:

Symptoms:

- **Headache**
- **Fever (+dates)**
- **Neurology (+dates)**
- **Rash (type)**

Vaccination Hx: (Specifically TBE, JE, Dengue, YF):

Imaging (MRI, CT):

EEG:

LP results (WCC/protein/glucose)

- **CSF WCC (differential especially lymphocytes):**
- **Local CSF viral panel result:**
- **Local CSF MC&S result:**

Local teams working diagnosis:

The above information is required for clinical diagnosis. Please could you also send us samples ***Insert sample type*** if available.

Kind regards

Clinical Team, RIPL
